# Influence of *Epilobium parviflorum* Herbal Extract on Physicochemical Properties of Thermoplastic Starch Films

**DOI:** 10.3390/polym16010064

**Published:** 2023-12-24

**Authors:** Magdalena Zdanowicz

**Affiliations:** Center of Bioimmobilisation and Innovative Packaging Materials, Faculty of Food Sciences and Fisheries, West Pomeranian University of Technology, Szczecin, Janickiego 35, 71-270 Szczecin, Poland; mzdanowicz@zut.edu.pl

**Keywords:** *Epilobium parviflorum*, food packaging, hoary willow herb, thermoplastic starch

## Abstract

In this study, for the first time, *Epilobium parviflorum* Schreb. (E, hoary willowherb) aqueous extract was introduced into edible biopolymer films and its influence on physicochemical properties of the final products were investigated. Potato starch was gelatinized in the herbal tea to obtain thermoplastic starch (TPS) films via the casting method. The characterization of the films included mechanical, antioxidative, water (WVTR, contact angle, swelling degree) and UV radiation barrier properties as well as microstructure analysis (SEM). Obtained results indicated that the presence of the extract (rich in phenolic compounds) in the films acted as a co-plasticizer for starch and led to a higher elongation at break, up to 70%, with a parallel increase in tensile strength up to ca. 9 MPa. Moreover, TPS films with E exhibited lower WVTR values and absorption of UV light in comparison with the control TPS film. DPPH scavenging activity of TPS E films immersed in methanol was ca. 92%, and it was related to the release of the extract into liquid media. Novel TPS E films are characterized by multifunctional properties that can be used, e.g., in the active packaging sector.

## 1. Introduction

According to data from 2015 [1], 49 M tons of plastics were used for food packaging (international production of plastics was 322 M tons). The most common polymers applied in this field of the industry are polyolefins and polyesters like poly(ethylene terephthalate). However, despite their low cost and performance, they are ballast for the environment, due to their lack of biodegradability. Global waste plastic generation by the packaging sector was 141 M tons/year [2]. Moreover, many packaging products are blends, composites or laminates and consist of a few polymeric materials, which impedes their recycling. Application of biodegradable and natural-based materials like polysaccharides, proteins and polyesters synthetized from natural compounds like polylactide or polyhydroxyalcanoates isolated from bacteria is one of alternatives for fossil fuel-based plastics [3]. Polysaccharides like starch, chitin and cellulose are abundant biopolymers used in different sectors of industry. A widely abundant and cheap starch, it is easy to extract from plant sources (e.g., corn, potatoes or cassava), and after gelatinization in an aqueous system with a plasticizer presence (e.g., polyols), it exhibits good film-forming properties without the necessity of complex modification, for example, via derivatization, in comparison with cellulose (only some derivatives such as carboxymethyl, methyl or hydroxypropyl methyl cellulose are water-soluble) or chitin (which needs deacetylation to chitosan, which is soluble in an acidic environment). Native starch is sensitive to water and microbial activity, thus, its utilization for packaging purposes is limited. Nanobio-composites can be formed [4], or functional additives like essential oils [5] or plant extracts can be added to improve microbial resistivity as well as other features like barrier properties (against UV radiation, moisture or oxygen) and mechanical properties. Herbal extracts, due to their abundance, non-toxicity and low costs, are widely studied as functional additives for polymers (both oil-based as well as those of natural origin) intended for food packaging [6,7].

The hoary willowherb (*Epilobium parviflorum* Schreb.)—E—is the herbaceous perennial plant of the Onagraceae family. This plant occurs in most parts of Europe, Northern Africa and Western Asia. Willowherb tea exhibits anti-inflammatory, diuretic and antioxidative properties. It is used for gastrointestinal and prostate disorders, mucous membrane lesions and improving wound healing [8]. Epilobium species contain phenolic acids (e.g., gallic acid, chlorogenic acid, (Z)-p-coumaric acids), tannins, flavonoids (e.g., myricetin, catechin, koempherol), steroids and terpenes [9]. Depending on the herb species and plant parts, the content of polyphenols in lyophilizate from an aqueous solution is 9–19%, along with tannins 6–32% and flavonoids [10]. Sixteen volatile compounds of E tea infusion were identified via HS-SPME chromatography in Bajer et al.’s work [11]. Of the volatile profile, 22.5% was dodecanol, monoterpenes were about 10%, monoterpenoids were about 8%, and the phenolic derivatives estragole and E-anethole were about 14%. The extracts from plants belonging to the Epilobium genus are considered nontoxic [12].

The literature presents more works related to other herbs from the family *Epilobium agnustifolium* Schreb. that exhibit similar properties (antiaging, anti-inflammatory or antioxidative properties [13,14,15,16,17,18]) to *Epilobium parviflorum*, but the latter is more popular in Poland. This herb is more available and easier to purchase as a pure herb or part of different supplements, especially those intended for the prostate and urinary tract. In the literature, there is no information about the influence of the willowherb’s presence in biopolymeric films on their properties. There are in fact only a few works related to polysaccharides and the herb extract. In work [19], microcrystalline cellulose and its silicate derivative were used as a macromolecular adjuvant for the production of tablets with E extract that exhibited, for example, a short dissolution time. In Egil and colleagues’ study [20], E extract was loaded in chitosan nanoparticles prepared with ionic gelation. These two works showed some investigation of the interactions between polysaccharidic carrier agents and E extracts but in the context of the pharmaceutical application.

The aim of this work was to investigate willowherb extract’s presence on TPS film properties. Physicochemical (tensile, barrier and antioxidative, color, morphology and microstructure) properties were studied. Total phenolic compound (TPC) content was determined both for the herbal tea as well as for the films. The influence of E extract on biodegradable, edible films’ properties was examined using tensile machine, FTIR and SEM analysis, and the moisture content water vapor transmission rate (WVTR), swelling degree and surface contact angle were measured. The results of this study on biopolymeric films with the herb addition have been described in the literature for the first time.

## 2. Materials and Methods

### 2.1. Materials

Native potato starch (29 wt% amylose content, 15.5 wt% moisture) as a film-forming material was supplied by Nowamyl S.A., hoary willowherb *Epilobium parviflorum* (E) was purchased from Dary Natury (Grodzisk, Poland) and glycerol (anhydrous) used as a plasticizer was obtained from Chempur (Piekary Śląskie, Poland). DPPH–(2,2-diphenyl-1-picrylhydrazyl radical) (Tokyo Chemical Industry Co., Tokyo, Japan) and methanol (pure, Chempur) were used for the evaluation of antioxidative properties. Folin-Ciocalteau reagent (Eurochem BGD, Tarnów, Poland), gallic acid (p.a. 97.5–102.5%, Sigma-Aldrich, Milan, Italy) and Na_2_CO_3_ (anhydrous, reagent grade, Scharlau, Barcelona, Spain) were applied for the measurement of total phenolic compounds content in the extract and the films.

### 2.2. Hoary Willowherb Tea Preparation

The main extract, 2 wt% of dry herb, was dispersed in distilled water and stirred (500 rpm) in a sealed glass beaker for 1 h at 60 °C on a magnetic stirrer. Then, the aqueous extract was filtrated first with mesh to separate the herb solid parts and then filtered through paper filter to remove the remains. The filtrate was used as an aqueous media for starch gelatinization and further film formation. To obtain 1 wt% extract, the main extract was diluted. The total phenolic content in the tea was determined and is described in Section Determination of TPC in *Epilobium parviflorum* Tea.

### 2.3. TPS Film Preparation

Glycerol (25 pph on dry starch) and starch were added to the herbal tea to obtain 5 wt% of starch dispersion. The systems were placed in a water bath and stirred for 30 min at 90 °C. Gelatinized starch systems were poured on polystyrene Petri dishes and dried for 3 h at 55 °C in the dryer and then placed in the Binder climate chamber (Tuttlingen, Germany) and dried overnight at 45 °C and 50% RH. Dried films were stored in a climate room at 25 °C and 50% RH for 1 week before testing. Moisture content of the samples after conditioning was measured using a drying balance (MA 110.R, RADWAG, Radom, Poland). TPS E1 is a sample with the extract from 1 g of hoary willowherb (1 wt%), and TPS E2 is a sample with the extract from 2 g (2 wt%) of herb per 5 g of dried starch. Reference sample TPS without extract was prepared in an analogue way, but distilled water was used instead of herbal tea.

### 2.4. Characterization of TPS Films

#### 2.4.1. Mechanical Tests

Mechanical tests were performed using a Zwick/Roell Z2.5 tensile tester (Ulm, Germany). The films were cut into 5 mm wide strips; the initial grip separation was 25 mm, and the cross-head speed was 10 mm/min. At least six replicate samples were tested. The elongation at break (EB), maximum tensile strength (TS) and Young’s modulus (YM) with standard deviations were calculated with TestXpert II software.

Statistical analysis of the mechanical test data for the extruded samples was subjected to a one-way analysis of variance and the significant difference was determined by the significance difference test (*t*-Student’s test).

#### 2.4.2. Fourier Transform Infrared Spectroscopy (FTIR)

For FTIR analysis, a Perkin Elmer Spectrophotometer (Spectrum 100, Waltham, MA, USA) using the ATR technique was applied. The spectra were analyzed in wavenumber range 4500–600 cm^−1^ with 32 scans using OMNIC software (Ver. 7.3).

#### 2.4.3. UV-Vis Spectrophotometric Analysis of TPS Films

The UV-Vis spectrophotometer UV-Vis Thermo Scientific Evolution 220 (Waltham, MA, USA) was applied for film analysis in the wavelength range of 190–800 nm. The transparency of the films was determined based on transmittance value at 700 nm [21].

#### 2.4.4. Water Vapor Transmission Rate (WVTR)

WVTR determination was performed using the gravimetric dish method ISO 2528 [22] at 23 °C and 75% RH for 24 h. For the measurements, 68-3000 EZ-Cup Vapometers (Thwing-Albert Instrument Company, West Berlin, NJ, USA) were applied. Granular silica gel was used as a desiccant, and a minimum of three replicates were tested. Films were conditioned at 23 °C and 75% RH for 24 h before WVTR determination. The parameter calculations were based on the slope/diameter of the sample × 24 h.

#### 2.4.5. Determination of Swelling Degrees

Swelling degrees (SD%) were determined as in previous work [23]. Samples (20 × 20 mm), which had been dried overnight at 55 °C before tests, were immersed in distilled water for 24 h. Then, the swollen samples were placed on a paper towel for 10 min to remove excess water and were weighed. SD was calculated according to the following equation: SD = (m_s_ − m_d_/m_d_)100%; where m_s_—mass of swollen sample, m_d_—mass of dried sample.

#### 2.4.6. Contact Angle Measurements

Surface contact angle determination of TPS films for water was performed using an SEO contact analyzer Phoenix-Mini (PM-041807, Suwon, Republic of Korea). Contact angle values were calculated using Surfaceware 8 software after distilled water drop deposition.

#### 2.4.7. Determination of Total Phenolic Content (TPC) and the Compounds’ Migration into Tested Liquid

##### Determination of TPC in *Epilobium parviflorum* Tea

In this study, the Folin–Ciocalteau method was applied with gallic acid as an equivalent according to the Singleton and Rossi method [24]. First, 5 mL of aqueous extract, 75 mL of distilled water and 5 mL Folin–Ciocalteau reagent and saturated Na_2_CO_3_ solution were added to the volumetric flask (100 mL), and distilled water was topped up to the flask’s mark. Then, samples were kept for 1 h in a dark place. The absorbance of the samples was measured using Spectrophotometer Evolution 220 UV-Visible (Thermo Scientific, Waltham, MA, USA) at 760 nm wavelength. Results are presented as gallic acid equivalent (mg GAE/L) of the phenolic compounds.

##### Determination of TPC Migrated from TPS Films

The direct method of evaluation of TPC in TPS films was a test of phenolic compound migration into aqueous media. First, 0.25 g of cut films (10 × 10 mm) were placed in 100 mL volumetric flasks that were filled up to the mark. Then, volumetric flasks were placed in an ultrasound bath and kept there for 30 min. Thereafter, samples were kept for 30 min at 25 °C. The TPC determination in the aqueous extractant was conducted as for the extract described above.

#### 2.4.8. Determination of Antioxidative Properties of Willowherb Tea and TPS Films

The ability of herbal tea active compounds and the films with their presence to scavenge DPPH (2,2-diphenyl-1-picrylhydrazyl radical) was studied. Films were cut to 20 × 20 mm parts placed in falcons, 1 mL of water was added along with 3 mL of methanol and 1 mL of methanolic solution of DPPH and then the samples were kept for 10 min in a dark place. The solution from the stored samples was measured immediately using a spectrophotometer UV-Vis (Evolution 220 UV-Visible Spectrophotometer, Thermo Scientific), at a wavelength of 517 nm. The degree of scavenged radicals was calculated according to the equation DPPH% = (A_DPPH_ − A_s_) × 100%/A_DPPH_, where A_DPPH_ is absorbance of the control sample and A_s_ is absorbance of the studied sample with the film.

#### 2.4.9. Color Determination of TPS Films

The CIELAB color scale was determined with a Colorimeter CR-5 (Konica Minolta, Tokyo, Japan). Samples were measured in 5 repetitions at random locations on each studied TPS film. Moreover, chroma (c*), which is assigned to the gray color and the impurity of the film, was calculated according to the following equation:

$$(c^{*})=\sqrt{\left[{\left(a^{*}\right)}^{2}+{\left(b^{*}\right)}^{2}\right]}$$

#### 2.4.10. Scanning Electron Microscopy

The starch-based films were analyzed using a scanning electron microscope (SEM) Vega 3 LMU microscope (Tescan, Brno-Kohoutovice, Czech Republic). The test was carried out at 25 °C with a tungsten filament and an accelerating voltage of 10 kV was used to capture SEM images. All specimens were viewed from above.

## 3. Results and Discussion

### 3.1. Mechanical Test Results

**Figure 1:**
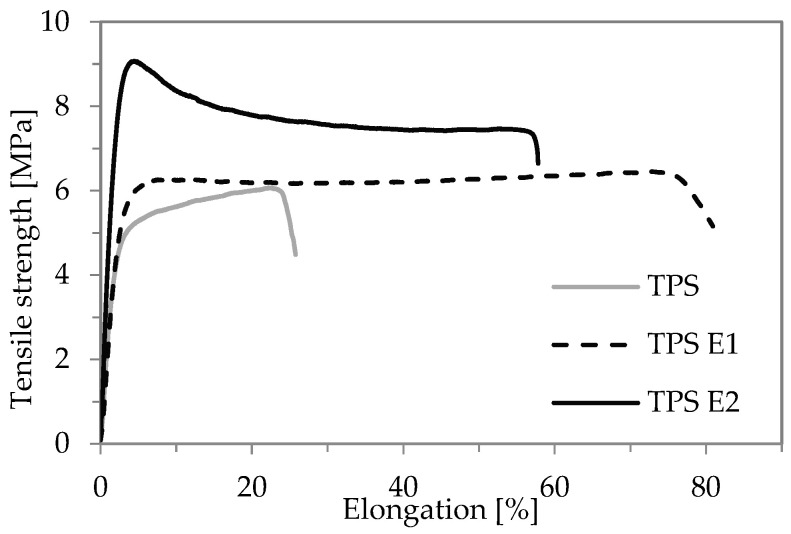
Stress-strain curves of TPS films.

Mechanical test results are presented in Table 1 and Figure 1. It was found that E’s presence improved the mechanical properties of TPS films.

The higher the content of the herbal extract, the higher are the TS and YM values of TPS. For TS for TPS E2, the value is 50% higher (8.2 MPa) than TPS (6.0 MPa). Interestingly, after the extract addition, not only TS increased but also EB increased, and for TPS E1, the increase was twofold higher (70%) than for the reference sample (35%). This indicates that E extract had an extra plasticizing effect on the gelatinized starch matrix without having a negative effect on TS as in the case of conventional plasticizers, where a higher plasticizer content leads to higher EB but lower TS [25]. The co-plasticization in TPS E1 and E2 is depicted in Figure 1. This can be caused by the presence of large molecules of polyphenols that are strongly joined by hydrogen bonding (a more noticeable field point on stress-strain curves), but not so strong as to restrain polysaccharide chain mobility assigned to higher elongation. In work [26], higher EB for starch films with Hibiscus sabdariffa extract was explained also by the disruption of the semi-crystallinity of the polysaccharide caused by the formation of that H-bonding. Interestingly, in our work, an improvement in mechanical properties was obtained without an effect of decreasing EB, whereas starch films with other polyphenol-rich extracts exhibited the opposite behavior. In the work of Pinẽros-Hernandez [27], cassava starch films with 10% aqueous rosemary extract had higher TS, but EB was three times lower than in the case of the reference sample. Starch-based films with grape cane extract [28], cocoa nibs [29] and betel leaf extract [30] exhibited higher EB but lower TS. Starch with green tea polyphenols exhibited lower TS and EB than TPS without additives [31]. In the literature, there are not many works where the improvement of mechanical properties (higher both TS and EB) was obtained. Among them are works that presented studies on yucca starch/alginate films with mesocarp beet and black eggplant extract [32] and corn starch with rice straw extract [33].

### 3.2. FTIR Spectroscopy

**Figure 2:**
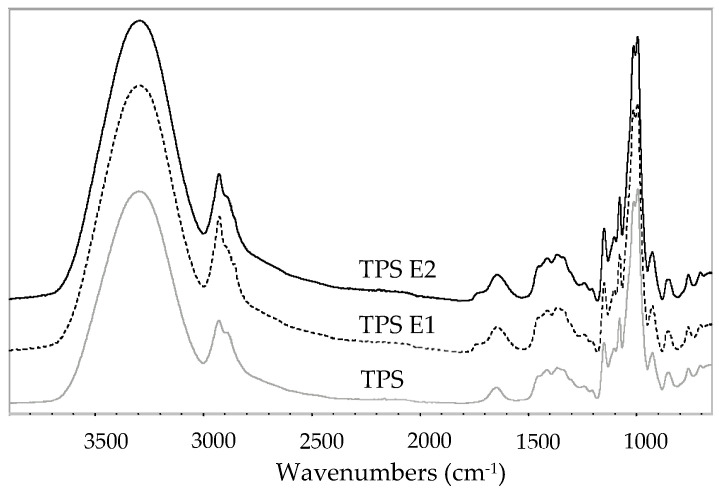
FTIR-ATR spectra of the films.

Figure 2 shows FTIR-ATR spectra of all the TPS films. The broad band in the range of ca. 3620–3000 cm^−1^ for all the TPS films was assigned to stretching free, inter- and intramolecular hydroxyl groups. The band at the range of 3000–2800 cm^−1^ is related to stretching C-H bonds. There is a shift of the peak into a lower wavenumber from 3294.5 cm^−1^ for TPS to 3290.7 and 3288.7 for TPS E1 and E2, respectively. This can be caused by the H-bonding formation between compounds from the extract with TPS. This H-bond crosslinking can also be responsible for changes in mechanical properties (higher TS and YM) [34]. Fingerprint peaks for the polysaccharide are in the range of 1200–900 cm^−1^ and are assigned to stretching C-O and C-C bonds as well as bending C-O-H groups from anhydroglucoside units of the polymer. There are no qualitative differences between the spectra of the reference sample and TPS with the extract, except the bands with low intensity at 1733 cm^−1^ for TPS with E. This band comes from the E extract and is assigned to C=O in acetyl groups from hemicellulose [35,36] present in the herb. Some microfibers or microparticles of hoary willowherb residues could migrate through a sieve to the aqueous extract and remain in the system with gelatinized starch. This band can be also observed for TPS with grape cane extract [28] or with yerba mate tea [37], although it was not discussed in these papers.

### 3.3. UV-Vis Spectrophotometric Analysis of TPS Films

**Figure 3:**
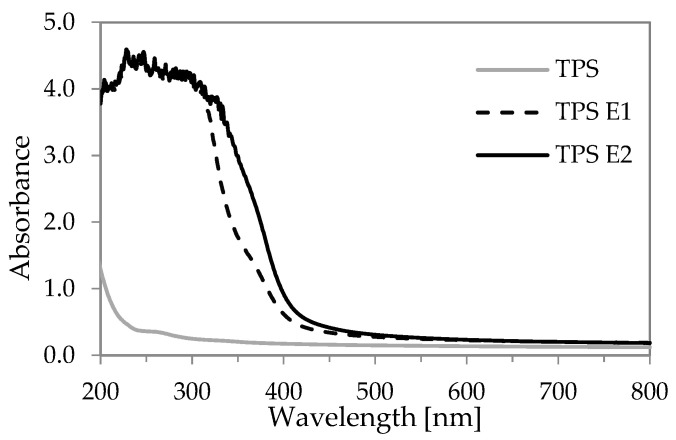
UV-Vis absorbance for TPS films.

UV-Vis spectra of the TPS films are presented in Figure 3. TPS films with the presence of E extract exhibit a wide band in a range of 190–380 nm. This indicates that polyphenol-rich E extract in the film absorbs UV light in its whole range. This phenomenon is related to the presence of π conjugated systems with hydroxyl-phenolic groups (that are present e.g., in phenolic acids and flavonoids in the extract) that absorb UV light [38]. High barrier properties for UV light can be used in functional food packaging such as foils or coatings protecting food products that are e.g., sensitive to photooxidation against organoleptic changes. Similar properties were obtained for other modified TPS films with plant extracts; absorbed UV-A light was obtained with, for example, adzuki bean starch films with cocoa nibs extract [29], sago TPS films with betel leaf extract [30], cassava starch with black tea [39] and wheat protein isolate films with *Epilobium agnustifolium* extract [18]. All these additives are known as products containing different phenolic compounds.

### 3.4. Behavior in Water, WVTR and Surface Contact Angle of TPS Films

WVTR and swelling degrees (SD) are listed in Table 2. Data indicate that the introduction of hoary willowherb extract into TPS improved moisture barrier properties and decreased SD. WVTR dropped from 524 g/m^2^·24 h for the reference TPS to 425 g/m^2^·24 h for TPS E2. The decrease in moisture permeability can be related to the presence of polyphenolic compounds in the polymer matrix bonded with polysaccharide chains with H-bonding. These results are correlated with the data obtained from mechanical tests. Moreover, some hemicellulose residues (as FTIR results showed, see Section 3.2) can affect permeability of the films. Such improvement in barrier properties can be obtained for starch-based films with tannins [40], aqueous green tea extract [41] and basil leaves extract [42]. Interestingly, water vapor permeability (WVP) values for TPS with extracts from betel leaves [43] and rosemary [27] were similar or even higher than TPS without additives. The TPS surface contact angle values were lower for the films with the extract and corresponded with the results obtained from WVTR determination. However, low values of the parameter in range of 37–50° indicate the highly hydrophilic character of the samples’ surface.

### 3.5. Phenolic Compounds’ Migration from the Films

**Figure 4:**
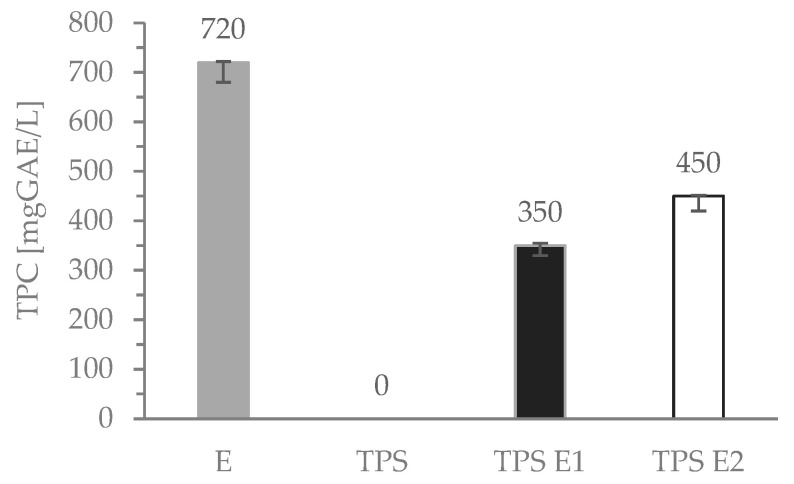
Total phenolic content in hoary willowherb tea and TPS films.

Total phenolic content was determined for the liquid media in which TPS and TPS E films were stored (see Section Determination of TPC Migrated from TPS Films). The results are presented in Figure 4. The TPC for the pure E extract is 720 mgGAE/L (for 2% of the herb in water), and for TPS E1 and TPS E2, it is 350 and 450 mgGAE/L, respectively. The higher content of the extract in the polysaccharide film, the higher the content of the released phenolic compounds into the test environment. This indicates that these active compounds are not strongly and covalently bonded with the polymeric matrix as the FTIR-ATR results showed.

### 3.6. Antioxidative Properties

Hevesi et al. [43] revealed that aqueous/acetone extract of E exhibited a higher antioxidant effect than well-known antioxidants (Trolox, ascorbic acid) and inhibited lipid peroxidation in small concentrations.

**Figure 5:**
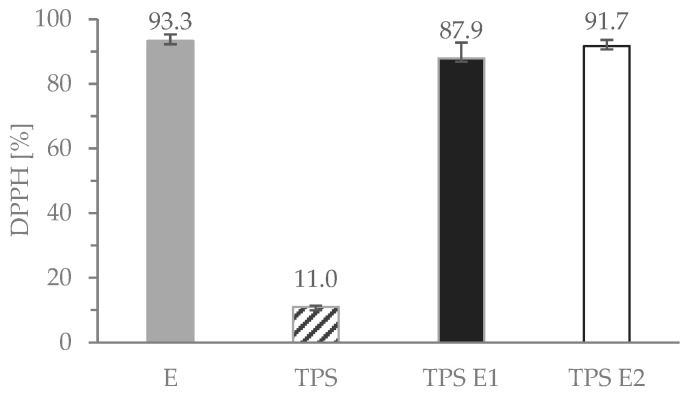
Antioxidative activity of TPS films in methanol.

Both herbal tea and modified TPS films exhibited high antioxidative properties (Figure 5).

For TPS E2, the DPPH scavenging activity reached 91.7%. These high values of DPPH% can be related to the high content of phenolic compounds (that can scavenge a reactive form of oxygen), that, due to not being strongly bonded in the starch matrix, retained their activity and were able to “sweep” the free radicals. Moreover, the nature of the TPS matrix, due to the hydrophilicity and quite high swelling degree (Table 2), can facilitate migration of the additive into the environment. High values of DPPH% for the control TPS film can be attributed to abundant hydroxyl groups [44], e.g., from glycerol, that can also migrate from the film into the liquid (methanol). High antioxidant activity of the studied samples is similar or even quite higher than, e.g., TPS with grape cane extract [28], rosemary extract [45], Chinese bayberry extract (rich in anthocyanins) [44] or even gallic acid [46].

### 3.7. Color of TPS Films

Table 3 shows values of L*a*b* of CIELAB three-dimensional color space. L* is lightness (black at 0, white at 100), a* is the green-red opponent color axis and b* is assigned to yellow-blue opponent colors (both scales from 60 to −60).

TPS films are slightly opaque and semi-transparent. The introduction of E into TPS films led to a decrease in the transparency and lightness of the films. Due to the herbal tea color, using herbal tea instead of water dyed the starch films a yellow color (high b* values). The higher content of the extract, the darker and more intense yellow color of the TPS films, as b* and c* parameters indicated. A low minus value a* for films with extract suggested a slightly green tint in the yellow color of the samples.

### 3.8. Surface Morphology Analyzed with Scanning Electron Microscopy (SEM)

**Figure 6:**
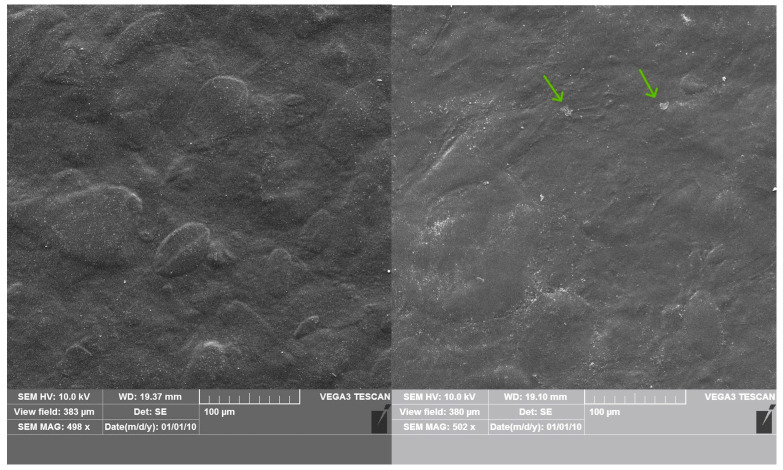
SEM micrographs of TPS (**left**) and TPS E2 (**right**) films with 500× magnitude. Green arrows point out the E herb remaining.

The surface microstructure of TPS without and with the extract was compared using SEM (Figure 6). The film obtained from starch gelatinized in water only exhibited an inhomogeneous surface where the granules’ shape remnants called “ghosts” [47] can be observed. This evidences that the films were obtained via a gelatinization process that involved the granules’ swelling not dissolution. This morphology can result in a slightly opaque appearance of the films (Table 3). On the other hand, films obtained from starch that was gelatinized in the herbal tea have a smoother surface than the control film. This indicates that hydrogen bonds between swelling starch, glycerol and compounds from the extract can be formed during the gelatinization process, restricting further retrogradation of the polysaccharide. In TPS E films, small particles (ca. 10 nm) can be noticed (green arrows on Figure 6). These particles, coming from the herb’s parts, were also detected with FTIR-ATR (rich in hemicellulose) (Figure 2). Higher tensile strength and Young’s modulus and lower WVTR, swelling degree and moisture content and contact angle and transparency can be a result of their presence in the polymeric materials.

## 4. Conclusions

The addition of *Epilobium parviflorum* (E) aqueous extract to biodegradable natural source-based films has not been presented in the literature so far. Potato starch gelatinization in E tea significantly affected the properties of the final TPS films. The mechanical properties of TPS E films were improved (higher elongation at break, as well as tensile strength and Young’s modulus) in comparison with the reference sample without E, indicating some co-plasticizing effect of the extract’s presence. Moreover, the WVTR value was 24% lower for films containing the extract. The results from the mechanical test, FTIR, SEM and study of behavior in moisture and water show that not only H-bonding between E and TPS but also some hemicellulose remnants affected the physicochemical properties of the materials. The broad absorption band in the range of 190–380 nm on UV-Vis spectra for the TPS films with E extract indicates that the films with polyphenol-rich E extract absorbed UV light in the whole range. Active compounds from the herb yielded high DPPH scavenging activity (93.3%), i.e., TPS E2 exhibited almost 92%. SEM revealed that the surface of the films that were obtained via gelatinization in herbal tea was smoother than films obtained from starch gelatinized in distilled water. The results indicate that multifunctional properties of TPS films with willowherb extract can be applied in food packaging, for example, for active packaging prolonging the shelf life of many food items (especially for fatty or oily products).

## Figures and Tables

**Table 1:** Mechanical properties of TPS and TPS with *Epilobium parviflorum* Tea.

Sample	Mechanical Properties		
	TS [MPa]	EB [%]	YM [MPa]
TPS	6.0 (±0.87)	35 (±8.4)	225 (±12.0)
TPS E1	6.3 (±0.43)	70 (±16.0)	230 (±6.5)
TPS E2	8.7 (±1.00)	59 (±18.0)	285 (±11.2)

TS–tensile strength; EB–elongation at break; YM–Young’s modulus.

**Table 2:** Water and moisture barrier properties of TPS and TPS with *Epilobium parviflorum* Tea.

Sample	Behavior in Moisture and Water				
	WVTR Slope	WVTR_RH75%_ [g/m^2^·24 h]	Swelling Degree [%]	Contact Angle [°]	Moisture Content [%]
TPS	0.0692	524 (±0.4)	438 (±18.2)	36.9 (±5.51)	10.2 (±1.29)
TPS E1	0.0567	430 (±27.4)	355 (±17.6)	46.0 (±2.82)	8.8 (±0.38)
TPS E2	0.0561	425 (±15.2)	388 (±20.5)	50.5 (±5.68)	9.9 (±0.93)

**Table 3:** Color parameter values in L*a*b* scale.

Sample Acronym	L*	a*	b*	c*	Transparency T_700 nm_ [%]
TPS	94.2 (±0.09)	0.08 (±0.00)	0.28 (±0.08)	0.28	73 (±0.71)
TPS E1	93.4 (±0.21)	−2.0 (±0.06)	16.8 (±0.92)	16.9	64 (±1.41)
TPS E2	88.7 (±0.29)	−2.1 (±0.03)	29.6 (±0.79)	31.7	65 (±1.41)

## Data Availability

No new data were created or analyzed in this study. Data sharing is not applicable to this article.
